# Simultaneous measurement of intrinsic promoter and enhancer potential reveals principles of functional duality and regulatory reciprocity

**DOI:** 10.21203/rs.3.rs-6363545/v1

**Published:** 2025-05-13

**Authors:** Mauricio I. Paramo, Alden King-Yung Leung, Sagar R. Shah, Junke Zhang, Nathaniel D. Tippens, Jin Liang, Li Yao, Yiyang Jin, Xiuqi Pan, Abdullah Ozer, John T. Lis, Haiyuan Yu

**Affiliations:** 1 Department of Molecular Biology and Genetics, Cornell University; Ithaca, NY, USA; 2 Weill Institute for Cell and Molecular Biology, Cornell University; Ithaca, NY, USA; 3 Department of Computational Biology, Cornell University; Ithaca, NY, USA

## Abstract

Growing evidence indicates that transcriptional regulatory elements can exert both promoter and enhancer activity; however, the relationship and determinants of this dual functionality remain poorly understood. We developed a massively parallel dual reporter assay that enables simultaneous assessment of the intrinsic promoter and enhancer potential exerted by the same sequence. Parallel quantification for thousands of elements reveals that canonical human promoters and enhancers can act as both promoters and enhancers under the same contexts, and that promoter activity may be necessary but not sufficient for enhancer function. We find that regulatory potential is intrinsic to element sequences, irrespective of downstream features typically associated with distinct element classes. Perturbations to element transcription factor binding motifs lead to disruptions in both activities, implicating a shared syntax for the two regulatory functions. Combinations of elements with different minimal promoters reveal reciprocal activity modulation between associated elements and a strong positive correlation between promoter and enhancer functions imply a bidirectional feedback loop used to maintain environments of high transcriptional activity. Finally, our results indicate that the magnitude and balance between promoter and enhancer functions are shaped by both intrinsic sequence properties and contextual regulatory influences, suggesting a degree of plasticity in regulatory action. Our approach provides a new lens for understanding fundamental principles of regulatory element biology.

## Main

Cellular processes are precisely controlled through complex gene expression programs that integrate signals from gene-proximal promoters and gene-distal enhancers. Traditionally, promoters and enhancers have been considered separate classes of transcriptional regulatory elements (TREs), often distinguished by their functions, chromatin environments, and genomic locations. Increasing evidence, however, has revealed broad similarities between both the architecture and function of these two regulatory elements, complicating their classification as distinct types^1,2^.

Promoters are classically defined as DNA sequences that drive RNA polymerase II (RNAPII) transcription initiation at gene-proximal transcription start sites (TSS)^3^. The region around the TSS (typically defined as the ±50 base pair (bp) region surrounding the TSS) is known as the core promoter, which contains core promoter motifs, such as the Goldberg-Hogness box (TATA box) and the Initiator (Inr)^4^. These motifs are recognized and bound by general transcription factors (GTFs), such as TBP and TFIIB, that recruit and assemble the RNAPII pre-initiation complex (PIC). RNAPII transcription initiation, promoter-proximal pausing, and pause-release into productive elongation are further facilitated by gene-, cell-, and state-specific transcription factors (TFs) and co-factors that bind at the promoter and at other regulatory sequences such as enhancers to regulate gene transcription^5^.

Conversely, enhancers are defined as DNA sequences that stimulate transcription at a distance, irrespective of their position and orientation with respect to their target gene^6^. The first enhancer discovered was a 72 bp sequence from the SV40 genome that could dramatically increase the transcription of the *β-globin* gene in a distance- and orientation-independent manner^7–9^. Enhancers were soon after discovered in Mammalia^10^ and are now recognized as key elements in the regulation of eukaryotic transcription. Today, enhancers are generally thought of as TF-binding regulatory regions that come into proximity to their target gene promoters via three-dimensional DNA looping^11^.

Numerous findings now point to promoters and enhancers being far more similar than previously thought, spearheaded by the discovery of widespread transcription at enhancer loci^12,13^. Detailed characterization has revealed that when active, both are encompassed within nucleosome-depleted regions (NDRs), bound by sequence-specific TFs that facilitate the assembly of PICs at core promoter regions, and undergo divergent transcription initiation and pausing, with protein-coding gene promoters divergently transcribing mRNAs and upstream antisense RNAs (uaRNAs) in either direction and enhancers divergently transcribing enhancer RNAs (eRNAs) in both^14–19^. These remarkable similarities in chromatin and sequence architecture have led to a proposed unified model for transcriptional regulatory elements^2,14,16^.

Prior attempts at defining promoters and enhancers have largely assumed that their functions are distinct and non-overlapping. However, enhancers possess intrinsic promoter potential^12,13,20^, and numerous studies are now reporting that promoters can exert distal enhancer activity^21–24^. Understanding how these two activities within the same element relate and elucidating their shared determinants is necessary to ensure that current models of gene regulation capture the full complexity of regulatory element functional duality.

To date, experiments that measure both the promoter and enhancer potentials of the same set of sequences are lacking. One of the few studies to have employed such a strategy on a large scale was Nguyen et al., which used separate massively parallel reporter assays (MPRAs) to measure promoter and enhancer activities for the same elements^25^. This attempt, however, like all existing MPRAs, inherently decouples the intrinsic promoter and enhancer potential from one another, regulatory potentials that are likely interrelated in their native contexts. One method developed by Mikhaylichenko et al. detected promoter and enhancer activities simultaneously, though was limited to a small test set of elements given the utilization of fluorescent *in situ* hybridization (FISH) as their functional readout^26^.

For these reasons, we developed a Quantitative Unifying Assay for Simultaneously Active Regulatory Regions by sequencing (QUASARR-seq), a massively parallel dual reporter assay that allows for the simultaneous measurement of intrinsic promoter and enhancer potentials exerted by the same element, for thousands of elements in a single experiment. We perform systematic functional comparisons between intrinsic promoter and enhancer functions exerted by canonical promoter and enhancer elements, revealing a degree of entanglement between the two activities, across element types. Our findings suggest that regulatory potential is embedded within element sequences, with impairments in element promoter activity showing compounding effects by also impairing enhancer function. Finally, using combinations of elements with different minimal promoters (minPs), we find that paired elements exert reciprocal regulatory influences to establish promoter and enhancer functions of each element, and that promoter-enhancer activity balance is shaped by both intrinsic sequence properties and contextual regulatory influences, suggesting a degree of plasticity in regulatory action.

## Results

**Fig. 1:**
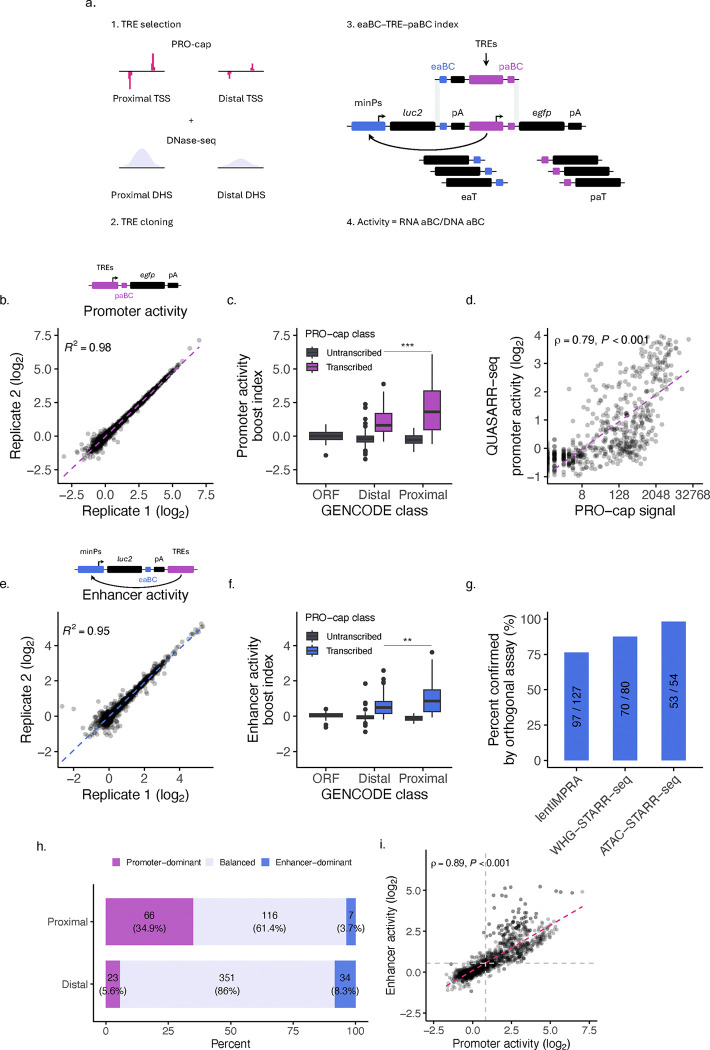
QUASARR-seq simultaneously measures an element’s intrinsic promoter and enhancer activity. **a**, Schematic of the QUASARR-seq workflow. 1, Candidate active promoters were defined as PRO-cap proximal TSSs, active enhancers as distal TSSs, inactive promoters as PRO-cap untranscribed, DNase-seq proximal DHSs, and inactive enhancers as untranscribed distal DHSs (see **Methods**). 2, The cloning of TREs was carried out as previously described^27^. 3, High-complexity eaBC-TRE-paBC indices for accurate and robust measurements are achieved through the generation of eaBC-TRE-paBC amplicons via consecutive isothermal oligo extensions. The QUASARR-seq vector features a minimal promoter (minP) that drives the expression of a reporter gene (*luc2*) that contains a degenerate 20 bp enhancer activity barcode (eaBC) located within the 3′ untranslated region (UTR) of an enhancer activity transcript (eaT). Candidate TREs are cloned downstream of this reporter system such that they can drive the expression of a second reporter gene (*egfp*) that contains another degenerate 20 bp promoter activity barcode (paBC) located within the 5′ UTR of a promoter activity transcript (paT). Each reporter system is insulated with a cleavage and polyadenylation site (pA) to avoid confounding the two readouts. 4, QUASARR-seq quantifies activity as the ratio of aBC RNA transcripts to aBC DNA input, normalized using negative controls (see **Methods**). **b**, Correlation of QUASARR-seq promoter activity measurements between replicates. **c**, Promoter activity boost indices of elements parsed by GENCODE and PRO-cap class (*P*-value < 0.001, Student’s t-test). **d**, Correlation between QUASARR-seq promoter activity measurements and PRO-cap signal (Spearman’s *ρ* = 0.79, *P*-value < 0.001). **e**, Correlation of QUASARR-seq enhancer activity measurements between replicates. **f**, Enhancer activity boost indices of elements parsed by GENCODE and PRO-cap class (*P*-value < 0.01). **g**, Percent confirmed of QUASARR-seq active enhancers by lentiMPRA, WHG-STARR-seq, and ATAC-STARR-seq. Calculated based on 50% reciprocal overlap between elements across assays. **h**, Activity balance category distribution by GENOCDE class. **i**, Correlation between QUASARR-seq measured promoter and enhancer activities (Spearman’s *ρ* = 0.89, *P*-value < 0.001). For box plots, center line represents median, while box limits indicate upper and lower quartiles. Whiskers extend to 1.5 × interquartile range, and points beyond whiskers denote outliers. Significance between groups was assessed using Student’s t-test, with significance levels indicated by asterisks (*P < 0.05, ** *P* < 0.01, *** *P* < 0.001).

To investigate the relation and determinants of promoter and enhancer element dual functionality, we sampled a set of TREs in the human immortalized myelogenous leukemia cell line K-562. We previously demonstrated that capped nascent RNA sequencing provides high predictive performance using 500 bp distance cutoffs from GENCODE annotations to distinguish promoter and enhancer elements (see **Methods**)^27^. Thus, we selected candidate active promoters defined as PRO-cap proximal TSSs and candidate active enhancers as distal TSSs (Fig. 1a). Additionally, we selected a set of candidate inactive promoters defined as PRO-cap untranscribed, DNase-seq proximal DNase I hypersensitive sites (DHSs) and candidate inactive enhancers as untranscribed distal DHSs.

Hereafter, for clarity, we use the terms ‘promoter activity’ to refer to the candidate TREs ability to drive transcription locally (i.e. initiation at the TRE) and ‘enhancer activity’ to refer to the candidate TREs ability to drive transcription from a distance (i.e. initiation at the minP).

### QUASARR-seq simultaneously measures an element’s intrinsic promoter and enhancer activity.

To systematically evaluate the functional duality of transcriptional regulatory elements, we developed a Quantitative Unifying Assay for Simultaneously Active Regulatory Regions by sequencing (QUASARR-seq), a massively parallel dual reporter assay that enables the simultaneous measurement of intrinsic promoter and enhancer potentials exerted by the same regulatory element molecule (Fig. 1a). QUASARR-seq adopts the principles of classical reporter assays where candidate regulatory sequences are cloned into expression vectors but incorporates two functional reporter systems each used to independently quantify promoter and enhancer activity for the same sequence (see **Methods**). Enhancer activity measurements are obtained by the reporter system positioned upstream of the TRE that contains a minP that drives the expression of enhancer activity barcodes (eaBCs) located within the 3′ untranslated region (UTR) of an enhancer activity transcript (eaT). Similarly, promoter activity measurements are obtained by the reporter system positioned downstream of the TRE where the TRE itself drives the expression of promoter activity barcodes (paBCs) located within the 5′ UTR of a promoter activity transcript (paT). As in all MPRAs, QUASARR-seq quantifies activity as the ratio of counts of aBC RNA transcripts to aBC DNA input vectors, from which boost indices are calculated by basal expression level normalization using negative controls (see **Methods**).

QUASARR-seq promoter activity measurements [log_2_(RNA paBCs/DNA paBCs)] across replicates demonstrated a high degree of reproducibility (*R*^*2*^ = 0.98; Fig. 1b). As expected, promoter activity varied significantly across GENCODE classes, with PRO-cap transcribed proximal elements (i.e., active promoters) exhibiting the highest activity, followed by transcribed distal elements (i.e., active enhancers), and with untranscribed elements and negative control open reading frames (ORFs) exhibiting little or no activity, respectively (*P*-value < 0.001, Student’s t-test; Fig. 1c). Interestingly, many of the proximal elements with the highest promoter activities were found to correspond to the promoters of long non-coding RNAs (lncRNAs) and small nuclear RNAs (snRNAs). The top three included: 1. the divergent promoter for LINC01138 (lncRNA)/RNVU1–21 (snRNA) and ENSG00000224481 (uncharacterized lncRNA), 2. the promoter for RNVU1–2A (snRNA), and 3. the divergent promoter for SNHG1 (lncRNA) and SLC3A2 (protein coding).

Promoter activity measurements showed a good correlation with native genomic PRO-cap (Spearman’s *ρ* = 0.79, *P*-value < 0.001; Fig. 1d) and PRO-seq signals analyzed under a variety of criteria (**Supplementary Fig. 1**; see **Methods**), suggesting that QUASARR-seq captures a significant, though incomplete reflection of transcription initiation and elongation patterns observed in endogenous contexts, similar to other reports using transient systems^22,25^. We hypothesize that these differences may be due to the effects of enhancers and other regulatory elements acting on the TREs in the native loci while QUASARR-seq is capturing their intrinsic promoter activity. We observed a similar correlation with Survey of Regulatory Elements^28^ (SuRE; Spearman’s *ρ* = 0.52, *P*-value < 0.001; **Supplementary Fig. 2a**) to those from PRO-seq (**Supplementary Fig. 1**), despite the low 10% reciprocal overlap between elements tested in both assays.

QUASARR-seq enhancer activity measurements [log_2_(RNA eaBCs/DNA eaBCs)] across replicates also demonstrated a high degree of reproducibility (*R*^*2*^ = 0.95; Fig. 1e). Enhancer activity too varied across GENCODE classes, albeit to a lesser degree, with proximal elements exhibiting a dynamic range closer to that of distal elements (*P*-value < 0.01; Fig. 1f). Again, many of the proximal elements displaying the highest enhancer activities corresponded to lncRNA promoters. The top three included: 1. the promoter for ENSG00000266401 (uncharacterized lncRNA), 2. the divergent promoter for ENSG00000287126 (uncharacterized lncRNA) and RPS7 (protein coding), and 3. the divergent promoter for SNHG1 (lncRNA) and SLC3A2 (protein coding). These results agree with extensive reports finding high levels of enhancer activity mediated by the promoters of lncRNAs^24,29,30^.

QUASARR-seq active enhancer calls were reliably corroborated by orthogonal assays, with ATAC-STARR-seq (98.1%), WHG-STARR-seq (87.5%), and lentiMPRA (76.4%) all showing reciprocal active enhancer call rates greater than 75% (Fig. 1g). Similarly, QUASARR-seq reliably captured active enhancer calls made by orthogonal assays (**Supplementary Fig. 2b**). In all, QUASARR-seq enables the simultaneous quantification of both promoter and enhancer activities from the same element, uniquely positioning us to address long-standing questions about the dual functionality of transcriptional regulatory elements.

**Extended Data Fig. 1:**
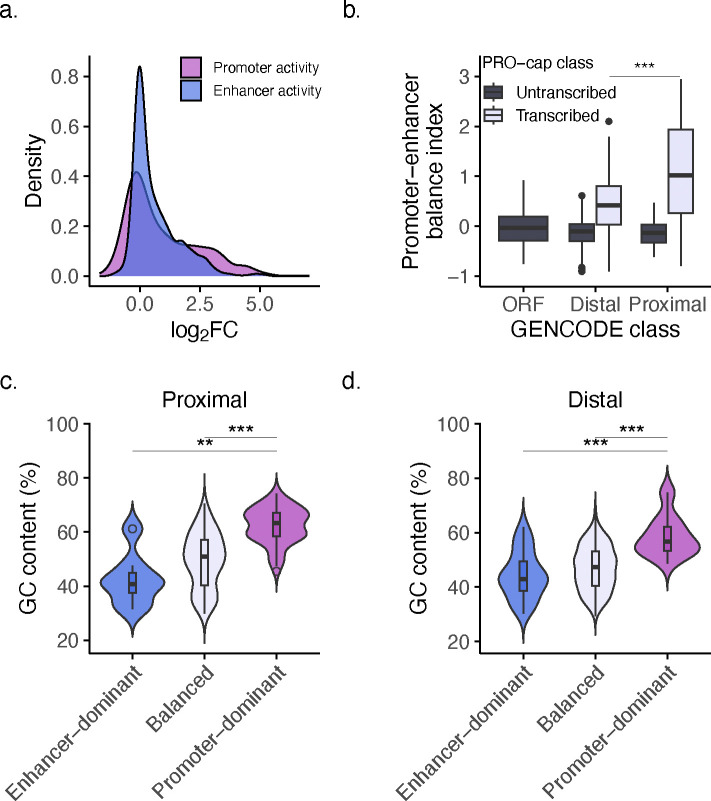
GC content drives functional bias in activity balance across GENCODE classes. **a**, QUASARR-seq promoter and enhancer Log_2_FC distribution. **b**, Ratio of promoter-to-enhancer activity boost index across GENCODE and PRO-cap classes (*p*-value < 0.001, Student’s t-test). **c-d**, GC content (%) of proximal (c) and distal (d) elements parsed by activity balance category (promoter-dominant vs. balanced for proximal and distal, *p*-value < 0.001; promoter-dominant vs. enhancer-dominant for proximal, *p*-value < 0.01; for distal, *p*-value < 0.001, Student’s t-test with Bonferroni correction). For box plot, center line represents median, while box limits indicate upper and lower quartiles. Whiskers extend to 1.5 × interquartile range, and points beyond whiskers denote outliers. Significance between groups was assessed using Student’s t-test, with significance levels indicated by asterisks (*P < 0.05, ** *P* < 0.01, *** *P* < 0.001).

To gain insight into the functional balance between activities, we calculated the balance index of promoter-to-enhancer activity boost indices (see **Methods**). This balance index was used to determine if an element predominantly acted as a promoter, an enhancer, or had a balanced dual function. Since promoter activity had a wider dynamic range than enhancer activity (Extended Data Fig. 1a), we Z-score normalized the two measurements prior to calculating the balance index. We defined “Promoter-dominant” as elements with a balance index > 1 (stronger promoter activity relative to enhancer activity), “Enhancer-dominant” as elements with a balance index < −1 (stronger enhancer activity relative to promoter activity), and “Balanced” as elements with a balance index falling between −1 and 1 (similar levels of promoter and enhancer activity, that is, a balanced function). We used these standard cutoffs as they corresponded to one standard deviation from the mean, highlighting elements with relatively strong activity dominance.

The number of elements in different balance index categories varied across GENCODE classes (Fig. 1h), with the distribution of balance indices differing significantly between proximal and distal elements (*P*-value < 0.001, Student’s t-test; Extended Data Fig. 1b). Proximal elements were mostly balanced (61.4%), but with a considerable number also displaying promoter-dominant behavior (34.9%). Interestingly, distal elements were predominantly balanced (86%), with very few enhancer-dominant (8.3%), indicating that promoter potential may be required for enhancer function. To investigate the potential drivers of functional bias in activity balance, we calculated the GC content (see **Methods**) of elements to compare between balance index categories. For both proximal and distal TREs, promoter-dominant elements were significantly more CG-rich than their balanced (proximal and distal, *P*-value < 0.001, Student’s t-test with Bonferroni correction) and enhancer-dominant (proximal, *P*-value < 0.01; distal, *P*-value < 0.001) counterparts (Extended Data Fig. 1c–d), suggesting that activity preference is, at least partially, sequence encoded.

### Promoter and enhancer activities are positively correlated.

Both positive and negative relationships between promoter and enhancer activities have been proposed^2,12^. According to the positive relationship hypothesis, the strength of an element’s promoter activity is directly related to its enhancer activity. In contrast, in the negative relationship hypothesis, the individual promoter activity strengths of interacting elements determine their respective enhancer activities following an inverse relationship, likely established by competition between the elements to determine their primary activity in a context-dependent manner.

Prior attempts at elucidating this relationship used decoupled promoter and enhancer activity measurements^25^, making the negative relationship hypothesis untestable as they could not capture the dynamic interplay between promoter and enhancer functions within the same element. Since the negative relationship hypothesis posits mutual exclusivity in promoter and enhancer activities, assessing their interrelation requires simultaneous measurement of both functions. To address this limitation, we utilized activity measurements obtained by QUASARR-seq, as it uniquely allows us to perform an agnostic comparison between these two competing models. QUASARR-seq measured promoter and enhancer activities across TREs were substantially positively correlated (Spearman’s *ρ* = 0.89, *P*-value < 0.001; Fig. 1i), more so than prior reports that yielded similar results^25^. These data support a model where the ability to drive transcription locally (i.e. promoter activity) may serve as a proxy for the ability to drive transcription from a distance (i.e. enhancer activity).

**Extended Data Fig. 2:**
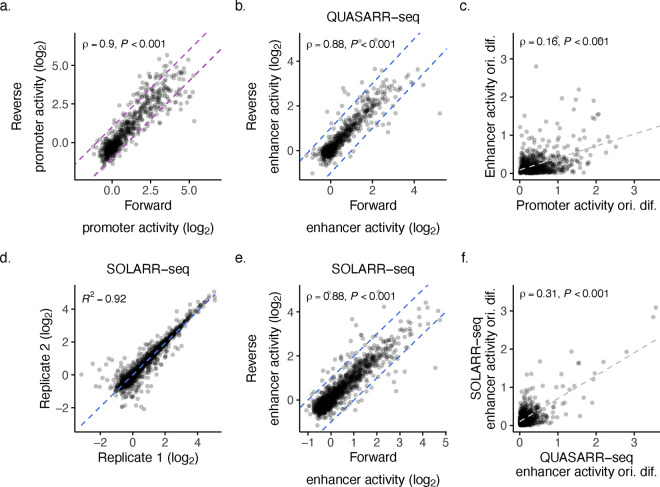
QUASARR-seq promoter and enhancer activity measurements are capturing related, but intrinsically decoupled regulatory functions of the same elements. **a**, Correlation of QUASARR-seq promoter activity measurements between elements cloned in forward and reverse orientations (Spearman’s *ρ* = 0.9, *P*-value < 0.001). **b**, Correlation of SOLARR-seq enhancer activity measurements between replicates. **c**, Correlation of QUASARR-seq enhancer activity measurements between elements cloned in forward and reverse orientations (Spearman’s *ρ* = 0.88, *P*-value < 0.001). **d**, Correlation of QUASARR-seq orientation difference between promoter and enhancer activities (Spearman’s *ρ* = 0.16, *P*-value < 0.001). **e**, Correlation of SOLARR-seq enhancer activity measurements between elements cloned in forward and reverse orientations (Spearman’s *ρ* = 0.88, *P*-value < 0.001). **f**, Correlation of enhancer activity orientation differences between of QUASARR-seq and SOLARR-seq (Spearman’s *ρ* = 0.31, *P*-value < 0.001).

Despite each reporter system being insulated with a cleavage and polyadenylation site (pA) to avoid confounding the two activity readouts (Fig. 1a; see **Methods**), we asked whether the high correlation between promoter and enhancer activities that we observed may be due to measuring the same function, perhaps caused from pA read-through transcription. To examine this, we used the activity measurements for elements cloned in forward and reverse orientations. We reasoned that if the promoter and enhancer readouts were measuring the same activity, then a clear relationship would be evident in the difference between their respective orientation activities. For both promoter and enhancer readouts, we found a strong correlation between activities when measured in both directions (Spearman’s *ρ* = 0.9, *P*-value < 0.001 and *ρ* = 0.88, *P*-value < 0.001, respectively; Extended Data Fig. 2a–b). Despite this, we found a weak association (*ρ* = 0.16, *P*-value < 0.001; Extended Data Fig. 2c) between the difference in orientation activity for promoter and enhancer activity measurements, supporting the case that the high correlation of promoter and enhancer activities is not an artifact of measuring the same function.

As a positive control, we performed the same analysis by comparing the enhancer activity measurements obtained from QUASARR-seq to those obtained from the MPRA SOLARR-seq. SOLARR-seq enhancer activity measurements were found to have a highly similar correlation between activities in both directions (*ρ* = 0.88, *P*-value < 0.001; Extended Data Fig. 2e), to those from QUASARR-seq. As expected, we found a stronger association (*ρ* = 0.31, *P*-value < 0.001; Extended Data Fig. 2f) between the differences in orientation activity for the two readouts, as they both measured the same function. In all, these data indicate that QUASARR-seq promoter and enhancer activity measurements are capturing related, but intrinsically decoupled regulatory functions of the same elements.

### Promoter activity is necessary, but not sufficient for enhancer function.

While increasing evidence suggests that transcriptional regulatory elements can exert both promoter and enhancer activities, it is still unclear whether these functions can co-occur or are mutually exclusive under the same regulatory contexts. Since QUASARR-seq measures both activities exerted by the same sequence simultaneously, we asked whether elements with measured promoter activity were also capable of being assayed for enhancer function.

**Fig. 2:**
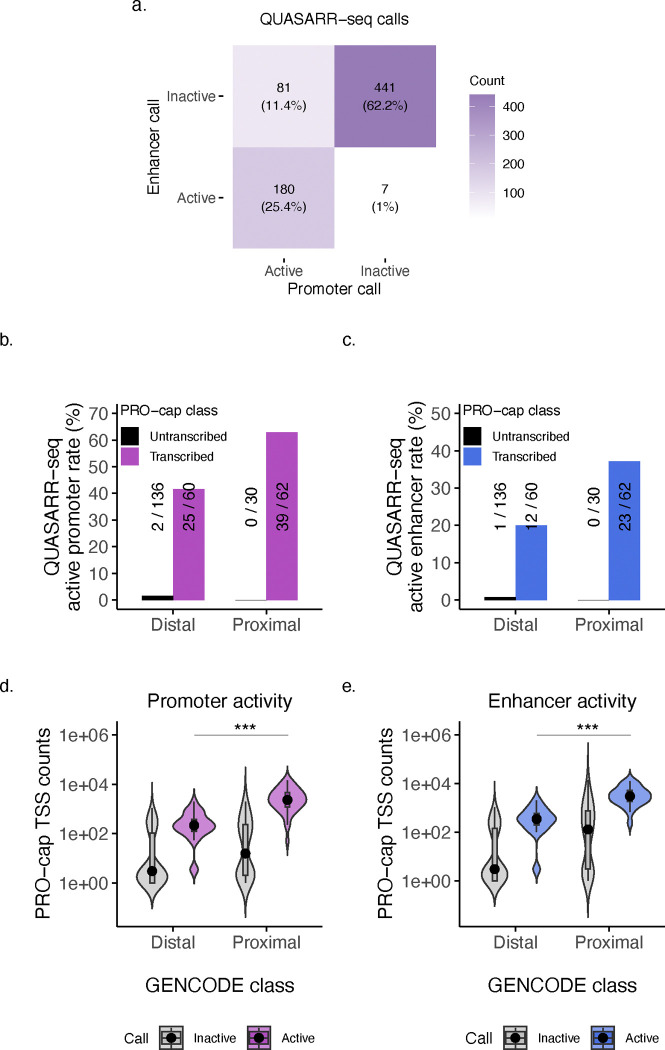
Promoter activity is necessary, but insufficient for enhancer function. **a**, Heatmap showing overlap between QUASARR-seq promoter and enhancer active element calls. Only elements with paired promoter and enhancer activity measurements obtained from the same experiment are included in this analysis. **b-c**, QUASARR-seq active promoter (b) and enhancer (c) rates by GENCODE and PRO-cap class. **d-e**, Element PRO-cap TSS counts by GENCODE class and QUASARR-seq active promoter (d) and enhancer (e) call (*P*-value < 0.001, Student’s t-test).

To investigate the concurrence of activities, we used a uniform processing pipeline (see **Methods**) to obtain QUASARR-seq-derived promoter and enhancer calls to make comparisons between activities. For this analysis, we used only data where we had paired promoter and enhancer activity measurements for the same element from the same experiment. Of the QUASARR-seq-called active promoters, we found that 69% (180/261) were also called active enhancers (Fig. 2a). More strikingly, of the QUASARR-seq-called active enhancers, 96.3% (180/187) were also called active promoters. These results suggest that promoter and enhancer activities can co-occur within the same regulatory context and that promoter activity may be necessary, but not sufficient for enhancer function.

### Promoters and enhancers can perform both promoter and enhancer functions.

We next asked whether QUASARR-seq active calls would stratify elements based on their canonical classifications. While almost all QUASARR-seq active promoters were PRO-cap transcribed elements, not all PRO-cap transcribed elements were deemed QUASARR-seq active promoters (Fig. 2b). This suggests that QUASARR-seq exhibits a high degree of specificity, with some reduction in sensitivity, which we hypothesize may be due to capturing the element’s promoter activity without the effects of other regulatory elements acting on them at their native loci. Similarly, essentially all QUASARR-seq active enhancers were PRO-cap transcribed (Fig. 2c), in line with our previous report that showed that transcription serves as a robust predictor for active enhancer elements^27^.

Both canonical promoters and enhancers could perform both promoter and enhancer functions. Still, active promoter calls varied across GENCODE classes, with proximal elements displaying the highest active rate (62.9%; Fig. 2b), followed by distal elements (41.7%). We observed a similar trend with active enhancer calls, with proximal elements exhibiting the highest active rate (37.1%; Fig. 2c), followed by distal elements (20%).

To explore potential drivers of differences in promoter and enhancer activity rates across GENCODE classes, we calculated the total PRO-cap read counts within the boundaries of tested elements. We hypothesized that the disparity in activity rates might reflect the inherent ability of proximal elements to initiate transcription at higher frequencies than distal elements. Supporting this hypothesis, we found that proximal elements generally exhibited higher endogenous initiation frequencies compared to distal elements (*P*-value < 0.001, Student’s t-test; Fig. 2d–e). Moreover, all active promoter and enhancer elements trended toward the upper bound in TSS counts.

In all, these data strengthen previous associations between transcription and enhancer activity. We find compelling evidence that essentially all active elements, independent of traditional classifications, require some intrinsic ability to initiate transcription. This suggests a functional entanglement in both activities, present across element types.

### Downstream sequence features do not confer element activity or type.

**Fig. 3:**
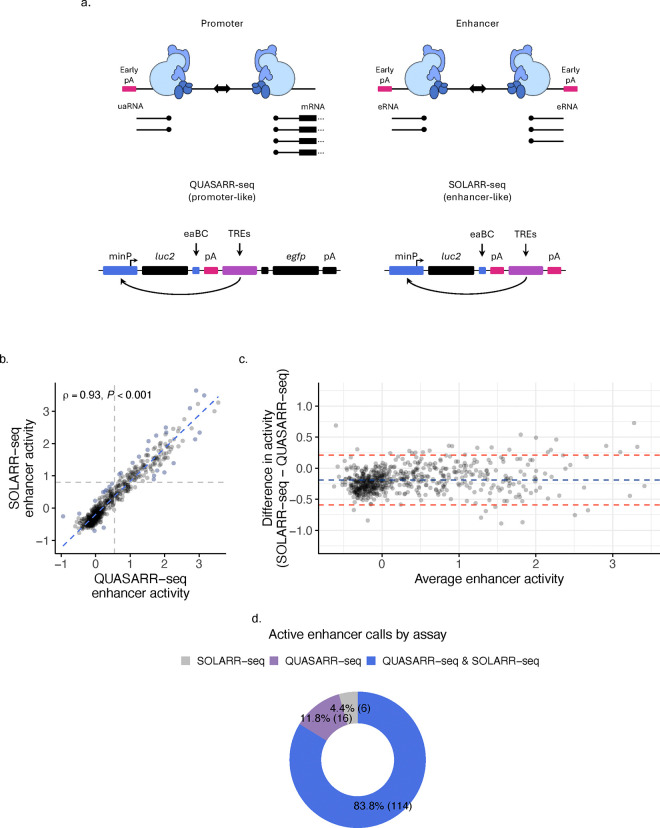
Downstream sequence features do not confer element activity or type. **a**, Schematic showcasing differences in downstream sequence features between canonical promoters and enhancers. QUASARR-seq mimics downstream features to those of promoters while SOLARR-seq mimics downstream features to those of enhancers. **b**, Correlation between QUASARR-seq and SOLARR-seq measured enhancer activities (Spearman’s *ρ* = 0.93, *P*-value < 0.001). **c**, Bland-Altman plot of agreement showing differences in activity measurements between SOLARR-seq and QUASARR-seq. **d**, Active enhancer calls by QUASARR-seq and SOLARR-seq.

Despite overwhelming similarity in chromatin and sequence architecture, the RNA transcripts produced by promoters and enhancers exhibit striking differences in their properties^20,31–33^ (Fig. 3a). mRNAs are typically long, spliced, and polyadenylated, whereas eRNAs are short, unspliced, and non-polyadenylated. Extensive evidence suggests that these differences in RNA classes are largely determined by the sequences present at the 5′ ends of transcripts (i.e., immediately downstream of the elements). Despite being non-polyadenylated, utilization of early poly(A) site sequences (e.g., AT/ATAA) is a discerning feature of eRNA transcription, which contributes to their short length and reduced half-life due to rapid degradation by the exosome complex^34^. In contrast, mRNAs are enriched with U1 small nuclear ribonucleoprotein (snRNP) recognized 5′ splice sites and are depleted of early poly(A) sites, where early U1 binding appears to impede early termination^35–38^.

It has been hypothesized that the early termination signals employed during eRNA transcription might contribute to enhancer function by promoting RNAPII turnover, which, presumably, could facilitate the transfer of transcriptional machinery from the enhancer to its associated promoter. In contrast, the absence of early termination signals and the presence of 5′ splice sites in mRNAs may inhibit transcriptional termination, thereby supporting efficient local elongation.

To test the hypothesis that downstream sequence features mediate *enhancerization* of regulatory elements, we compared enhancer activity measurements for the same set of TREs using two assays configured to simulate promoter and enhancer downstream regions. Since QUASARR-seq is designed to measure the intrinsic promoter activity of TREs, its paT is modeled to mimic attributes of mRNAs (Fig. 3a). In contrast, we developed Surveying OLigos for Active Regulatory Regions and sequencing (SOLARR-seq), a massively parallel reporter assay identical in sequence to QUASARR-seq except for the absence of the paBC and *egfp* components used for promoter activity measurements. Notably, SOLARR-seq incorporates early pAs immediately downstream of the TRE cloning cassette, mimicking attributes of enhancer downstream regions. We hypothesized that if early termination stimulated enhancer activity, a significant difference in activity would be observed between the two assays’ enhancer activity measurements.

SOLARR-seq enhancer activity measurements between replicates demonstrated a high degree of reproducibility (*R*^*2*^ = 0.92; Extended Data Fig. 2d) with minimal orientation-dependent activity (Spearman’s *ρ* = 0.88, *P*-value < 0.001; Extended Data Fig. 2e). Interestingly, a strong positive correlation was observed between QUASARR-seq and SOLARR-seq enhancer activity measurements (Spearman’s *ρ* = 0.93; Fig. 3b), indicating that despite substantial differences in downstream features, the two assays provided comparable enhancer activity readouts.

To evaluate potential systematic biases, including mean differences and outliers, we performed a Bland-Altman analysis^39^. This revealed consistent agreement between the two assays across activity ranges, with most differences falling within the limits of agreement (Fig. 3c). Using a uniform processing pipeline (see **Methods**), to obtain active enhancer calls for both QUASARR-seq and SOLARR-seq, we found a substantial overlap of active enhancers identified by both assays (83.8%; Fig. 3d), and that both proximal and distal elements were capable of exerting this enhancer function (**Supplementary Fig. 3a**).

Altogether, these results indicate that the striking differences in downstream sequence features between promoters and enhancers do not appear to influence regulatory activity or play a role in establishing element type, suggesting that regulatory potential is likely encoded within element sequences.

### Likely pathogenic variants that impair promoter activity have compounding effects by also impairing enhancer activity.

To further investigate the determinants of promoter and enhancer dual functionality, we sought to test the hypothesis that regulatory potential is embedded within element sequences. In previous work, we demonstrated that deletion of TRE core promoter regions (defined as −35 to +60 bp from the TSS) significantly impairs, and often completely ablates enhancer function^27^. Complete deletion of these core promoter regions, which disrupts enhancer function, would almost certainly also disrupt promoter function. Thus, to examine whether impairment of an element’s promoter activity leads to corresponding impairment of its enhancer activity with greater precision, we first focused on testing the impact of variants with strong clinical/functional evidence for disrupting promoter activity.

The variant c.-192A>G (ClinVar ID: 243006; gnomAD: 0.007%) is a nucleotide substitution 192 bp upstream of the ATG translational start site of the *APC* promoter 1B region^40^. This variant has been observed in individuals with clinical features of gastric adenocarcinoma and proximal polyposis of the stomach. Functional studies have demonstrated a damaging effect via binding disruptions of the TF YY1 and impaired activity of the *APC* promoter in gastric and colorectal cancer cell lines. Promoter impairment is further supported by an allelic imbalance in patient blood cells that suggests decreased allele-specific expression *in vivo*. Based on these findings, this variant has been classified as ‘Likely Pathogenic’.

**Fig. 4:**
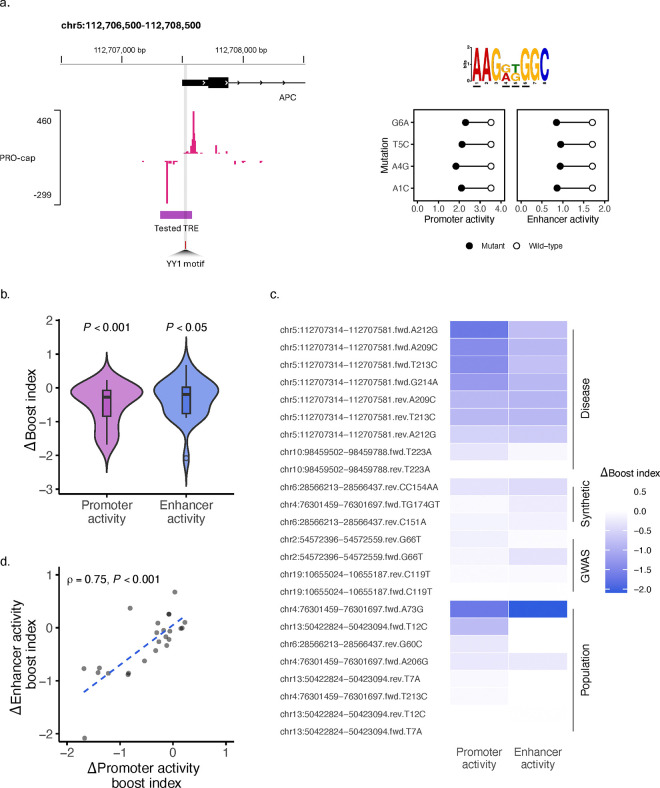
Promoter and enhancer effects are correlated in variants disrupting dual function. **a**, Left, Genome browser of the APC promoter locus that contains likely pathogenic variants. Top right, PWM logo of YY1 motif found in tested TRE. Bottom right. Lollipop plot showing wild-type (white circle) and mutant (black circle) allele promoter (left plot) and enhancer (right plot) activity measurements. **b**, Change (Δ) in promoter and enhancer activity boost indices between mutant and wild-type alleles (One-sample t-tests). **c**, Heatmap of Δ in promoter and enhancer activity boost indices for the disease-associated, synthetic, genome-wide association study (GWAS) identified, and population variants tested. **d**, Correlation of the Δ between promoter and enhancer activity boost indices (Spearman’s *ρ* = 0.75, *P*-value < 0.001).

In addition to c.-192A>G, hereafter referred to as A4G, we generated three additional variants (A1C, T5C, and G6A), each targeting a different base of the same YY1 motif (AAG[G/A][T/G]GGC) within the *APC* promoter (Fig. 4a). QUASARR-seq measurements of wild-type and mutant alleles of the element corroborated reported promoter activity impairment. More strikingly, simultaneous measurement of enhancer activity revealed a corresponding impairment on enhancer function. These results suggest that disruptions in an element’s promoter activity by likely pathogenic variants can have compounding effects by also disrupting its enhancer function. Dual impairment of regulatory activity could have broader implications in the way that damaging effects of disease-associated variants are identified or potentially treated.

### Promoter and enhancer effects are correlated in variants disrupting dual function.

To generalize these results, we expanded the set of elements to assess the effects of additional variants on dual regulatory function. We generated mutant elements containing disease-associated, synthetic, genome-wide association study (GWAS) identified, and population variants. In this analysis, we included only elements where we obtained both the promoter and enhancer activity of the wild-type and mutant alleles.

To assess the relation between impairments in dual regulatory function, we calculated the change (Δ) in promoter and enhancer activity boost indices by subtracting the wild-type element boost index from the mutant element boost index (see **Methods**). Here, a small Δ boost index indicated a large mutant disrupting effect. Variants reliably disrupted QUASARR-seq promoter activity measurements (*P*-value < 0.001, One-sample t-test; Fig. 4b). Moreover, we observed a similar trend with enhancer activity measurements, though with a lesser effect size compared to promoter activity (*P*-value < 0.05).

We next asked which mutation classes inflicted the largest disrupting effects on regulatory element activity. As expected, disease-associated variants generally imparted the smallest Δ in dual regulatory function (Fig. 4c), with a few population variants unexpectedly showing some high degree of disruption effects. We observed a trend between the Δ promoter and enhancer boost indices and found that both were substantially positively correlated (Spearman’s *ρ* = 0.75, *P*-value < 0.001; Fig. 4d). These results indicate that variants that disrupt an element’s promoter activity generally also disrupt its enhancer activity, suggesting a shared syntax for the two regulatory functions.

### Paired elements exert reciprocal regulatory influences to establish activities of both elements.

Recent studies propose a model in which an element’s enhancer activity is established by the quantitative tuning of the intrinsic promoter activity of its associated promoter, the intrinsic enhancer activity of the enhancer itself, and the three-dimensional contacts between the two, with TRE compatibility appearing to play an additional, though limited role^41^. It should follow then that an element’s promoter-enhancer dual functionality can be shaped by its interactions with other regulatory elements, such as its associated minimal promoter.

**Fig. 5:**
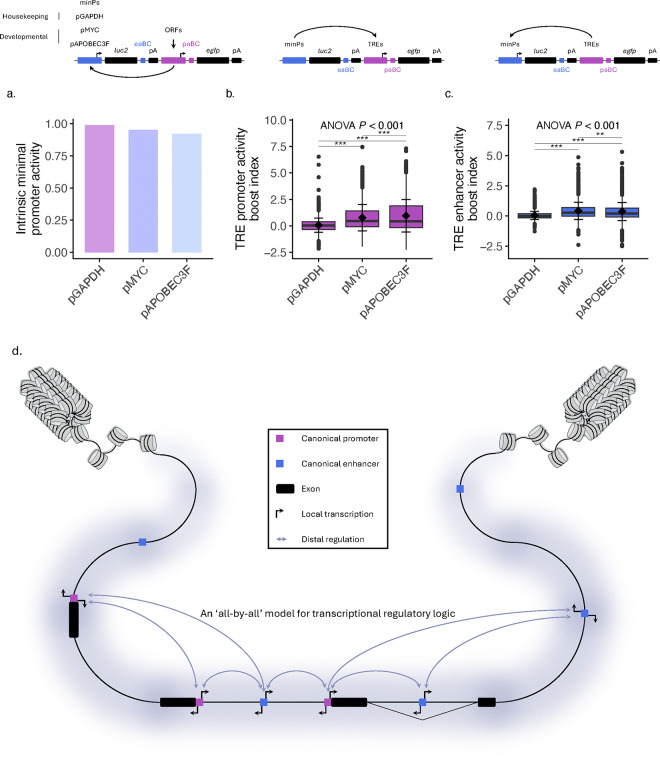
Paired elements exert reciprocal regulatory influences to establish activities of both elements. **a**, Left, QUASARR-seq pairing TREs with different minPs, including the promoters of a housekeeping gene; GAPDH, and a developmental gene; APOBEC3F, in addition to the promoter of MYC used thus far in this study. To distinguish between libraries, unique three-bp barcodes specific to each minP library were incorporated directly downstream of the paBCs. Right, Intrinsic promoter activities for the three minPs, calculated by taking their mean activities when paired with negative controls ORFs. Values shown are back-transformed from log_2_ scale. **b-c**, TRE promoter (b) and enhancer (c) activity boost index when paired with the minPs pGAPDH, pMYC, and pAPOBEC3F (Promoter activity, F(2, 8301) = 457.7, *P*-value < 2e-16, ANOVA; Enhancer activity, F(2, 8296) = 309.5, *P*-value < 2e-16; post-hoc analysis: all except pMYC vs. pAPOBEC3F for enhancer activity, *P*-value < 2e-16, Tukey’s HSD; pMYC vs. pAPOBEC3F for enhancer activity, *P*-value = 2e-07). **d**, An ‘all-by-all’ model for transcriptional regulatory logic. Three promoters (purple) and five enhancers (blue) are shown. Black blocks indicate exons. Diagonal lines indicate splice sites, used to denote intergenic and intragenic enhancers. Black arrows denote promoter activity, lavender arrows enhancer activity. Promoters and enhancers can exhibit functional duality. To exhibit dual functionality, promoter activity is necessary, but not sufficient for enhancer function. Promoters and enhancers can also exert regulatory reciprocity. Certain limitations, such as element compatibility or competition, may restrict associations and thus reciprocal regulatory modulation. Cartoon adapted from Andersson & Sandelin^1^ and Lenhard, Sandelin, & Carninci^3^. For box plots, center line represents median, while box limits indicate upper and lower quartiles. Whiskers extend to 1.5 × interquartile range, and points beyond whiskers denote outliers. Black diamonds represent mean activity, with error bars indicating ±1 standard deviation. Statistical significance was assessed using ANOVA followed by Tukey’s post hoc test, with significance levels indicated by asterisks (*P < 0.05, ** *P* < 0.01, *** *P* < 0.001).

To investigate the effects that different minimal promoters have on an element’s promoter and enhancer activities, we performed QUASARR-seq by pairing TREs with different minPs (Fig. 5a). For these experiments, we included the promoters of a housekeeping gene; *GAPDH*, and a developmental gene; *APOBEC3F*, in addition to the promoter of *MYC* used thus far in this study. To distinguish between libraries, we incorporated a unique three-bp barcode specific to each minP library positioned directly downstream of the paBCs, allowing us to identify the origin of each activity score and attribute it to its associated minP (see **Methods**). QUASARR-seq promoter activity measurements between replicates across minP libraries were highly reproducible (**Supplementary Fig. 4a-c**), with some reduction for enhancer activity measurements relative to promoter activity (**Supplementary Fig. 4g-i**).

Next, we calculated the basal promoter activities for the three minPs by taking their mean activities when paired with negative controls ORFs and found that the three were highly similar (Fig. 5a). Comparing activities across all minP sets, we observed a similarly strong positive correlation between promoter and enhancer activities (Spearman’s *ρ* = 0.75, *P*-value < 0.001; **Supplementary Fig. 5a**), suggesting that elements with higher promoter activity also generally tend to exhibit higher enhancer activity irrespective of their paired minP.

To gain insight into the impact that different minPs have on both the promoter and enhancer activities of the same element, we calculated the promoter and enhancer activity boost indices for each minP library (see **Methods**). Interestingly, pairing with different minPs had highly significant effects on both the promoter and enhancer activities of tested elements (Promoter activity, F(2, 8301) = 457.7, *P*-value < 2e-16, ANOVA; Enhancer activity, F(2, 8296) = 309.5, *P*-value < 2e-16; Fig. 5b–c). Post-hoc analysis revealed that elements paired with pMYC and pAPOBEC3F drove significantly higher promoter activity than when paired with pGAPDH, with a mean difference between pMYC and pGAPDH of 0.71 (*P*-value < 2e-16, Tukey’s HSD; Fig. 5b), pAPOBEC3F and pGAPDH showing an even greater difference of 0.89 (*P*-value < 2e-16), and a mean difference between pMYC and pAPOBEC3F showing a smaller but still significant effect of 0.18 (*P*-value = 2e-07). Similarly, Tukey’s HSD revealed that elements paired with pMYC and pAPOBEC3F exerted significantly higher enhancer activity than when paired with pGAPDH, with mean differences of 0.38 (*P*-value < 2e-16; Fig. 5c) and 0.32 (*P*-value < 2e-16), respectively, and with a difference between pMYC and pAPOBEC3F smaller at −0.06 (*P*-value = 0.002). These data suggest that reciprocal regulatory influences between associated elements together establish their respective functions.

Taken together, these data reveal a largely overlooked, though likely pervasive phenomenon in transcriptional regulation (Fig. 5d). Not only is the promoter activity of the target element (i.e., promoter) influenced by its associated elements (i.e., enhancers), but the promoter activity of the enhancers are also modulated by the target promoter. This dynamic interplay suggests that promoters and enhancers do not operate as independent unidirectional regulatory modules. Instead, promoters themselves exhibit enhancer-like functions to their enhancers (and likely other promoters), contributing to the system’s overall transcriptional potential, all while enhancers reciprocally influence the functional state of their target promoters (and likely other enhancers).

### Activity balance is shaped by intrinsic sequence properties and contextual regulatory influences.

**Fig. 6:**
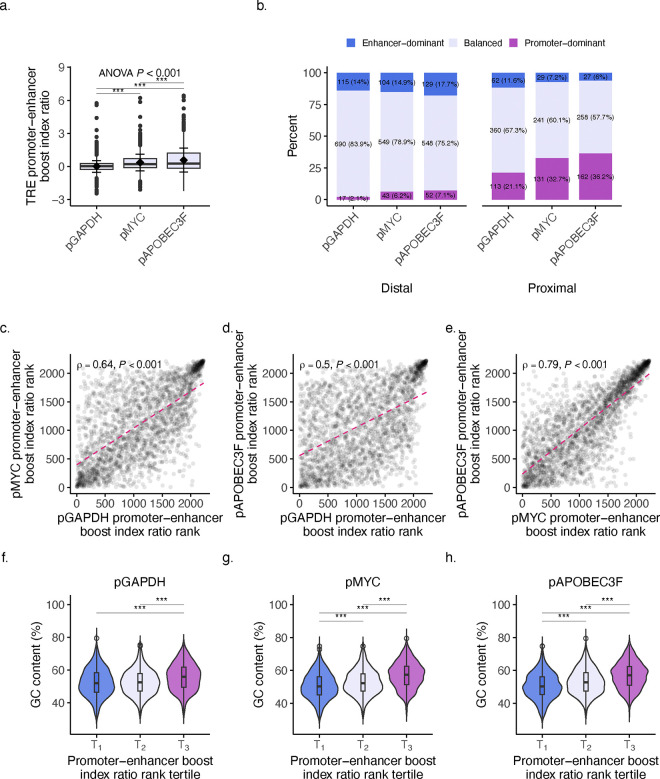
Activity balance is shaped by both intrinsic sequence properties and contextual regulatory influences. **a**, TRE promoter-enhancer activity boost index ratio when paired with the minPs pGAPDH, pMYC, and pAPOBEC3F (F(2, 8281) = 359.6, *P*-value < 2e-16, ANOVA; post-hoc analysis: *P*-value < 2e-16, Tukey’s HSD). **b**, Activity balance category distribution by GENCODE class when paired with the minPs pGAPDH, pMYC, and pAPOBEC3F. **c-e**, Correlation of boost index ratio ranks between minP libraries: pGAPDH vs. pMYC (Spearman’s *ρ* = 0.64, *P*-value < 0.001; c), pGAPDH vs. pAPOBEC3F (Spearman’s *ρ* = 0.5, *P*-value < 0.001; d), and pMYC vs. pAPOBEC3F (Spearman’s *ρ* = 0.79, *P*-value < 0.001; e). **f-h**, GC content (%) of elements paired with different minPs parsed by promoter-enhancer boost index ratio rank tertile: pGAPDH (f), pMYC (g), and pAPOBEC3F (h), Student’s t-test with Bonferroni correction. For box plot, center line represents median, while box limits indicate upper and lower quartiles. Whiskers extend to 1.5 × interquartile range, and points beyond whiskers denote outliers. Black diamonds represent mean activity, with error bars indicating ±1 standard deviation. Statistical significance was assessed using ANOVA followed by Tukey’s post hoc test, with significance levels indicated by asterisks (*P < 0.05, ** *P* < 0.01, *** *P* < 0.001).

We next asked what effect different minPs would have on the relationship between these two activities by examining the ratio of promoter-to-enhancer activity boost indices across minP sets (see **Methods**). This analysis revealed a significant effect of minP on activity ratio (F(2, 8281) = 359.6, *P*-value < 2e-16, ANOVA; Fig. 6a). Moreover, posthoc analysis showed that both pMYC and pAPOBEC3F led to significantly higher promoter-to-enhancer activity ratios compared to pGAPDH, with differences of 0.34 (*P*-value < 2e-16, Tukey’s HSD; Fig. 6a) and 0.57 (*P*-value < 2e-16), respectively, and with a difference between pMYC and pAPOBEC3F smaller at 0.23 (*P*-value < 2e-16). These findings suggest that minPs significantly influence the relative balance between promoter and enhancer activities, with elements paired with pAPOBEC3F and pGAPDH showing the highest and lowest activity ratios, respectively.

To gain insight into how different minPs influence the functional balance between promoter and enhancer activities, we calculated the balance index to categorize elements as promoter-dominant, enhancer-dominant, or balanced in function (see **Methods**). The distribution of elements across these categories varied between GENCODE classes, while also showing notable differences in distribution when paired with different minPs (Fig. 6b).

Among proximal elements, a striking shift was observed in the promoter-dominant category where 21.1% of elements were promoter-dominant when paired with pGAPDH, increasing to 36.2% when paired with pAPOBEC3F (Fig. 6b). This trend was accompanied by a decrease in the balanced category, from 67.3% with pGAPDH to 57.7% with pAPOBEC3F, suggesting that pAPOBEC3F stimulates element promoter activity preference at the expense of a more balanced function. The proportion of enhancer-dominant proximal elements was relatively low across all minPs, remaining around 6–12%.

For distal elements, the majority were categorized as balanced across all minPs, though the percentage decreased slightly from 83.9% with pGAPDH to 75.2% with pAPOBEC3F (Fig. 6b). Interestingly, the promoter-dominant category, which accounted for only 2.07% with pGAPDH, increased to 7.13% with pAPOBEC3F. The enhancer-dominant category also showed a modest increase with pAPOBEC3F (14.0% with pGAPDH vs. 17.7% with pAPOBEC3F). These results highlight that the pairing with different minPs significantly influences the functional dominance of elements, particularly in proximal regions, where pAPOBEC3F shifts the balance toward promoter-dominant activity. This suggests that minimal promoters can modulate the inherent regulatory balance of elements, potentially through differential compatibility between regulatory elements.

**Extended Data Fig. 3:**
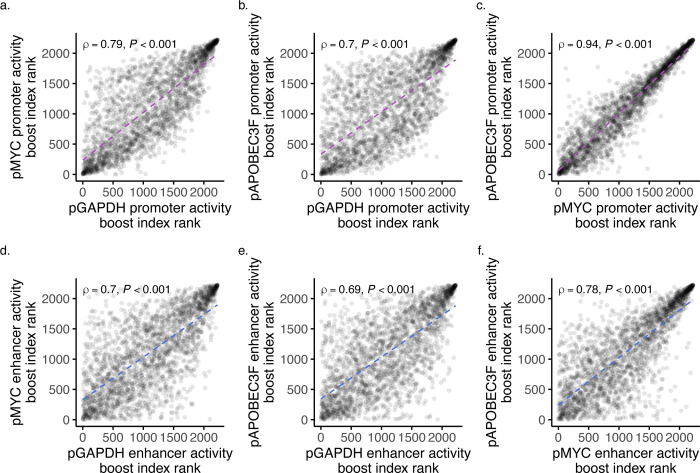
Comparisons of the different minP QUASARR-seq activity ranks. **a-c**, Correlation of promoter activity ranks between minP libraries: pGAPDH vs. pMYC (Spearman’s *ρ* = 0.79, *P*-value < 0.001; a), pGAPDH vs. pAPOBEC3F (Spearman’s *ρ* = 0.7, *P*-value < 0.001; b), and pMYC vs. pAPOBEC3F (Spearman’s *ρ* = 0.94, *P*-value < 0.001; c). **d-f**, Correlation of enhancer activity ranks between minP libraries: pGAPDH vs. pMYC (Spearman’s *ρ* = 0.7, *P*-value < 0.001; d), pGAPDH vs. pAPOBEC3F (Spearman’s *ρ* = 0.69, *P*-value < 0.001; e), and pMYC vs. pAPOBEC3F (Spearman’s *ρ* = 0.78, *P*-value < 0.001; f).

As a follow-up, we ranked the activity ratios for the three minP sets and revealed a consistent clustering at the extremes, with the highest and lowest ranked ratios showing agreement across sets (Fig. 6c–e). Notably, the variation in activity ratio ranks was distributed within these two extremes, suggesting that the intermediate-ranked elements (i.e., balanced elements) are most influenced by the swapping of the minP. Analysis of the individual activities revealed a similar pattern for promoter (Extended Data Fig. 3a–c) and enhancer activities (Extended Data Fig. 3d–f). This pattern may reflect the intrinsic properties of the elements themselves, such as GC-richness, where those with inherently high activity preference are less mutable by their associations with other elements. Conversely, elements with intermediate preference may be more susceptible to regulatory influence by other factors, such as associations with other elements. Interestingly, the strongest correlation in ratio ranks was observed between the two developmental promoters, pMYC and pAPOBEC3F (Spearman’s *ρ* = 0.79, *P*-value < 0.001; Fig. 6e), while the weakest correlation was found between the housekeeping promoter pGAPDH and the developmental promoter pAPOBEC3F (*ρ* = 0.5, *P*-value < 0.001; Fig. 6d).

To investigate the potential drivers of boost index ratio rank clustering, we calculated the GC content of elements and parsed them by rank tertiles. Visualization revealed that GC content imparted a significant influence on the rank clustering distribution (Fig. 6f–h). These results suggest an intrinsic biochemical foundation towards one activity over another. However, the activity balance of elements with intermediate GC content can be modulated by the regulatory context, particularly by the associated minP, which may stem from differences in element compatibility^42^.

### Minimal promoter modulation of activity balance reveals plasticity in regulatory function.

**Extended Data Fig. 4:**
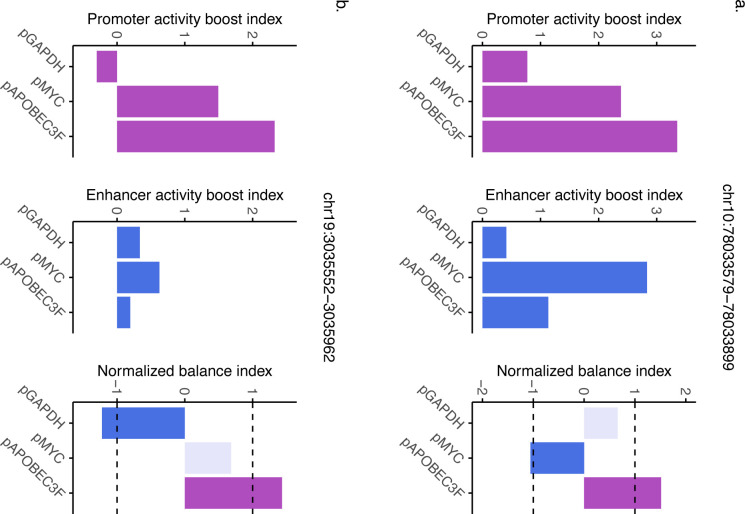
Minimal promoter modulation of activity balance reveals plasticity in regulatory function. **a-b**, Example of element shifting activity balance when paired with different minPs.

To further explore the dynamic nature of element dual function, we focused on cases where an element shifted between category extremes: changing from enhancer-dominant with one minP to promoter-dominant with another. While these cases were infrequent, their occurrence may underscore the plasticity of regulatory elements. One notable example was the TRE chr10:78033579–78033899, which exhibited balanced activity when paired with pGAPDH, enhancer-dominant activity with pMYC, and promoter-dominant activity with pAPOBEC3F (Extended Data Fig. 4a). Similarly, the TRE chr19:3035552–3035962 shifted from enhancer-dominant activity with pGAPDH to balanced activity with pMYC and finally to promoter-dominant activity with pAPOBEC3F (Extended Data Fig. 4b). These findings suggest that the regulatory function of these elements is not fixed but can be influenced by the minP they are associated with. This functional plasticity in regulatory action could reflect how elements may adapt their activity depending on different cellular or environmental contexts. In all, these data highlight the nuanced interplay between minPs and regulatory elements in shaping the balance between promoter and enhancer activities.

## Discussion

Our findings further challenge the conventional distinction between promoters and enhancers and instead support a continuum of regulatory potential present across element classes. Using QUASARR-seq, we demonstrate that both canonical promoters and enhancers can drive transcription locally and at a distance under the same regulatory contexts, finding evidence that promoter activity may be necessary, but not sufficient for enhancer function. This intrinsic dual potential suggests a unified mechanism rooted in sequence features and efficient factor recruitment.

The strong positive correlation between promoter and enhancer activities exerted by the same element could be indicative of a positive feedback loop, where reciprocal modulation between associated elements reinforces their regulatory functions. This bidirectional feedback may increase and maintain local concentrations of RNAPII, TFs, and co-activators to establish environments with high transcriptional activity. To this end, we propose an ‘all-by-all’ model for transcriptional regulatory elements (Fig. 5d), in which all elements (both canonical gene promoters and enhancers) exhibit functional duality to reciprocally regulate one another.

Our findings reveal that activity balance can be shaped by both intrinsic sequence properties and contextual regulatory influences. GC-rich sequences are linked to promoter-dominant behavior, suggesting an intrinsic biochemical foundation towards the one activity. However, the activity balance of GC-intermediate sequences can be modulated by the regulatory context, such as by its associated minP. For example, pairing with the developmental minP strengthened element promoter activity, while the housekeeping minP suppressed it. These results suggest some degree of plasticity in regulatory action. This adaptability may allow regulatory elements to shift their functional dominance in response to contextual cues, providing a robust mechanism for complex gene regulation in dynamic processes like development and differentiation.

Variants that disrupted promoter activity simultaneously impaired enhancer function, revealing the interconnected nature of these activities. This shared susceptibility has significant implications for understanding disease-associated variants as they may have compounding effects on gene regulation. These findings may underscore the need for therapeutic approaches that address the dual roles of regulatory elements.

While our study provides compelling evidence for a unified framework of TREs, we acknowledge several limitations. The transient nature of our assay may not fully recapitulate endogenous chromatin dynamics that likely influence regulatory interactions. Additionally, while we tested multiple minPs, expanding this repertoire could further elucidate the contextual factors that shape activity balance and regulatory reciprocity. Future work should move toward *in vivo* models and broader testing of TREs and minPs will be essential to validate and extend our findings. Still, we argue that a reductionist system that can isolate and dissect activities like QUASARR-seq is essential for elucidating fundamental principles of regulatory element logic as complex endogenous systems can be subjected to immense regulatory confounders.

To this end, our study advances our understanding of transcriptional regulation by demonstrating that promoters and enhancers may represent flexible functional states within a continuum. This paradigm shift provides new insights into the mechanisms of gene regulation and the functional consequences of regulatory variants in health and disease.

## Materials and Methods

### TRE definition and selection

To systematically compare promoter and enhancer elements, we defined candidate active promoters as PRO-cap proximal TSSs (distance cutoff <500 bp from GENCODE annotation), and candidate active enhancers as PRO-cap distal TSSs (distance cutoff >500 bp from GENCODE annotation). Similarly, we defined candidate inactive promoters as PRO-cap untranscribed, DNase-seq proximal DHSs (distance cutoff <500 bp from GENCODE annotation), and candidate inactive enhancers as PRO-cap untranscribed DNase-seq distal DHSs (distance cutoff >500 bp from GENCODE annotation). Transcribed elements were cloned using boundaries set at 60 bp downstream from each divergent TSS peak maximum. Untranscribed elements were cloned using boundaries set at DHS peak coordinates.

As negative controls, we included sequence-verified, TSS/DHS-screened human ORFs that showed no regulatory activity in eSTARR-seq^27^. As positive controls, we included a set of viral promoters/enhancers (*Cytomegalovirus* (CMV) and Rous sarcoma virus (RSV)), HS005^43^, and MYC E1–7^44^.

### TRE cloning

The cloning of TREs was carried out as previously described^27^. In brief, primers were designed in batch using our in-house web tool, which flanks forward primers with attB1′ and reverse primers with attB2′ 5′ overhangs. Primers used for TRE cloning were synthesized by Eurofins. K-562 genomic DNA (E493; Promega Corp.) was used as template for PCR amplifications using Phusion High-Fidelity DNA Polymerase (M0530; New England Biolabs) and PrimeSTAR GXL DNA Polymerase (R050A; Takara Bio Inc.). Amplicons were inserted into pDONR223 via Gateway BP cloning and single-colony-derived entry clones were sequence verified as previously described. Sequence-verified clones were propagated in lysogeny broth (LB) supplemented with spectinomycin, pooled, and purified using E.Z.N.A. Plasmid DNA Midi Kit (D6904; Omega Bio-tek, Inc.).

TREs were inserted into pDEST-luc-pMYC-preBC and pDEST-luc-pMYC-preBC-CCW via *en masse* Gateway LR cloning, propagated in LB supplemented with ampicillin and extracted using E.Z.N.A. Endo-Free Plasmid DNA Maxi Kit (D6926; Omega Bio-tek, Inc.). The resulting libraries were used as templates for downstream TRE barcoding (see eaBC-TRE-paBC index generation section).

### QUASARR-seq vector design and engineering

The QUASARR-seq vector design features an interchangeable minP that drives the expression of a reporter gene (*luc2*) that contains a degenerate 20 bp enhancer activity barcode (eaBC) in its 3′ UTR. Candidate TREs are cloned downstream of this reporter system such that they can drive the expression of a second reporter gene (*egfp*) that contains another degenerate 20 bp promoter activity barcode (paBC) in its 5′ UTR. Importantly, each reporter system is insulated with a cleavage and polyadenylation site (pA) to avoid confounding activity readouts from spurious initiation from the other element (i.e., initiation at the minP does not confound initiation at the TRE and initiation at the TRE does not confound initiation at the minP).

The QUASARR-seq assay vector, pDEST-QUASARR-luc-gfp-pMYC, was generated by modifying pDEST-luc-pMYC-preBC. To engineer pDEST-QUASARR-luc-gfp-pMYC, an egfp-pA reporter sequence was cloned downstream of the attR1-attR2 Gateway cloning cassette. We also generated the assay vectors pDEST-QUASARR-luc-gfp-pGAPDH, pDEST-QUASARR-luc-gfp-pAPOBEC3F, and pDEST-QUASARR-luc-gfp-pADGRG5 that are identical to pDEST-QUASARR-luc-gfp-pMYC except that the *MYC* minP was replaced with a *GAPDH*, *APOBEC3F*, or *ADGRG5* minP, respectively. minPs were cloned using boundaries set at 60 bp downstream from each PRO-cap-detected divergent TSS peak maximum.

pDEST-luc-pMYC-preBC was generated by modifying our eSTARR-seq assay vector, pDEST-hSTARR-luc-pMYC. To engineer pDEST-luc-pMYC-preBC, the attR1-attR2 cassette in pDEST-hSTARR-luc-pMYC was removed from the 3′ UTR and re-cloned downstream of the pA. To engineer pDEST-luc-pMYC-preBC-CCW, the attR1-attR2 cassette in pDEST-luc-pMYC-preBC was removed and then re-cloned back into its original position in reverse orientation.

The vectors pDEST-luc-pMYC-preBC and pDEST-luc-pMYC-preBC-CCW were used as surrogates to clone TREs in forward and reverse orientation, respectively and were used as templates for generating eaBC-TRE-paBC amplicons, which were subsequently cloned into the QUASARR-seq assay vector.

### eaBC-TRE-paBC index generation

Generation of high-complexity eaBC-TRE-paBC indices are required for accurate and robust enhancer and promoter activity measurements. High complexity maps were achieved through the generation of eaBC-TRE-paBC amplicons via consecutive isothermal oligo extensions using Phusion High-Fidelity DNA Polymerase (M0530; New England Biolabs). TREs were first cloned in both forward and reverse orientation into the surrogate vectors pDEST-luc-pMYC-preBC and pDEST-luc-pMYC-preBC-CCW, respectively. These libraries were used as templates for primer extension reactions using oligos that contain 1. 3′ ends complementary to TRE flanking sequences of the surrogate vectors, 2. internal 20 bp degenerate sequences, and 3. 5′ ends homologous to the QUASARR-seq assay vectors. Amplicons were cloned into pDEST-QUASARR-luc-gfp using NEBuilder HiFi DNA Assembly Master Mix (E2621; New England Biolabs), transformed, and plated on LB/agar supplemented with ampicillin. Colonies were scrapped into LB and extracted using E.Z.N.A. Endo-Free Plasmid DNA Maxi Kit (D6926; Omega Bio-tek, Inc.).

To ensure activity score accuracy and robustness and reduce potential eaBC/paBC-specific biases, a minimum of 10 eaBCs/paBCs should be uniquely assigned to each TRE. Thus, to obtain adequate eaBC-TRE-paBC coverage, the number of colonies required should exceed the product of the number of TREs being surveyed and the desired number of unique eaBCs/paBCs (i.e., surveying 100 TREs each with 100 eaBCs/paBCs requires a minimum of 10,000 colonies). To evaluate input library complexity and construct an eaBC-TRE-paBC index, pre-transfected eaBC-TRE-paBC libraries were generated prior to electroporation.

### Cell culture and nucleofection

K-562 (CCL-243; ATCC) cells were cultured in IMDM (30–2005; ATCC) supplemented with 10% FBS (30–2020; ATCC) at 37°C with 5% CO_2_. QUASARR-seq input libraries were electroporated into K-562 using Amaxa Cell Line Nucleofector Kit V (VCA-1003; Lonza Group AG) with a Lonza Amaxa Nucleofector II Device using program T-016. The manufacturers’ Amaxa Cell Line Nucleofector Kit V protocol for ATCC K-562 was followed, except for each electroporation, 1 × 10^6^ cells received 20 μg of QUASARR-seq library. Five electroporations were carried out per technical replicate, three technical replicates per biological replicate. Cells for different biological replicates were cultured and electroporated on separate days.

Following a 6-hour incubation period, cells were harvested by pooling samples for the same technical replicate to remove variance introduced during electroporation. Following three washes with 1X PBS (10010023; Gibco), each technical replicate was parsed out where 4 × 10^6^ cells were used for RNA libraries and 1 × 10^6^ cells were used for DNA libraries. Cell pellets were snap-frozen with LN_2_ and stored in −80°C until library preparation.

### QUASARR-seq library preparation

QUASARR-seq requires a minimum of four libraries per technical replicate: two RNA libraries targeting the eaBC and paBC transcripts, respectively, and two DNA libraries targeting the eaBC and paBC inputs, respectively. Total RNA was extracted from cells using TRIzol Reagent (15596026; Invitrogen) following the manufacturer’s instructions. Reverse transcription was performed with the total RNA as the template using SuperScript III Reverse Transcriptase (18080093; Invitrogen). Plasmid DNA was extracted from cells as previously described^45^. A first primer extension was performed with the extracted DNA as the template. Reactions were treated with Exonuclease I (M0293; New England Biolabs) to remove unused primer and purified using DNA Clean & Concentrator-5 (D4013; Zymo Research Corp.). A second primer extension was performed with the products of the reverse transcription (RNA libraries) and the first primer extension (DNA libraries) as the templates, respectively. Reactions were again treated with Exonuclease I and purified using DNA Clean & Concentrator-5. Finally, low-cycle PCR was performed to add sequencing adapters, followed by acquisition of 2 × 150 bp reads on an Illumina NovaSeq X Plus. All primer sequences used in this work can be found in **Supplementary Table 1**.

### QUASARR-seq data preprocessing

QUASARR-seq data preprocessing includes two main parts: 1. eaBC-TRE-paBC index mapping and 2. RNA aBC/DNA aBC activity measurement. Raw sequencing data was first filtered using fastp using the following parameters “--disable_adapter_trimming --trim_poly_g --cut_right”, followed by processing using biodatatools, with detailed commands and workflow found in the **Extended Text**. Briefly, sequence information was retrieved according to the library’s corresponding layout; eaBC-TRE-paBC and unique molecular identifiers (UMIs) for index mapping, and RNA aBCs, DNA aBCs, and UMIs for activity measurements.

In index mapping, eaBCs and paBCs were clustered by merging all sequences with a maximum Hamming distance of one for sequencing error tolerance. Partial element sequences were aligned to the reference element sequences. eaBCs and paBCs were then assigned to the TREs. For the multiple minP library set, an additional three-bp minP barcode was used to assign aBCs to their associated minPs. eaBCs and paBCs were regarded as a representation of a given TRE(A), if the number of eaBC-TRE(A)-paBC entries were significantly higher than all other eaBC-TRE(X)-paBC entries for any given TRE(X). In activity measurement, eaBCs and paBCs were extracted and matched to the previously clustered eaBCs and paBCs during index mapping and assigned to their corresponding TRE. All counts generated were subjected to UMI correction to collapse identical copies. The resultant RNA and DNA element counts were then normalized using edgeR to calculate the normalized element counts and logFCs.

### Activity boost index calculation

Activity boost index:

$$BI_{\mathrm{Promoter}}=\frac{A_{P}}{\mu_{P,\;\mathrm{ORFs}\;}}\;BI_{\mathrm{Enhancer}}=\frac{A_{E}}{\mu_{E,\;\mathrm{ORFs}\;}}$$

Where:

$A_{P}$ and $A_{E}$ are the measured promoter and enhancer activity logFCs, respectively.

$\mu_{P,\;\mathrm{ORFs}}$ and μE,ORFs are the mean promoter and enhancer activities of negative control ORFs, respectively.

### Uniform active call pipeline

To identify active TREs, we applied a uniform active element call pipeline, as previously described^27^. The pipeline begins with raw count matrices for DNA and RNA libraries. A pre-filtering procedure retains TREs that have counts per million (CPM) greater than a threshold calculated based on a raw count of 10 in the smallest DNA library. To account for library size differences and composition biases, a modified version of the trimmed mean of M values (TMM) normalization method^46^ was utilized to rely solely on negative control ORFs to normalize across libraries. log_2_-transformed RNA-to-DNA ratios (log_2_(RNA aBCs/DNA aBCs)) were computed as a measure of regulatory activity for each TRE using the limma-voom pipeline^47^. To assess enhancer activity, a Z-score approach was applied to compare the log_2_(RNA eaBCs /DNA eaBCs) values of each TRE to those of negative controls. TREs that have significantly higher regulatory activity than the basal transcription level defined by the negative controls in both orientations were identified as active enhancers. A similar approach was applied to identify active promoters.

### Activity balance index calculation

Z-score normalization:

$$Z_{\mathrm{Promoter}}=\frac{P\;-\;\mu_{P}}{\sigma_{P}}\;Z_{\mathrm{Enhancer}}=\frac{E\;-\;\mu_{E}}{\sigma_{E}}$$

Where:

P and E are the measured promoter and enhancer activity boost indices, respectively.

$\mu_{P}$ and $\mu_{E}$ are the mean activity boost indices for promoter and enhancer, respectively.

$\sigma_{P}$ and $\sigma_{E}$ are the standard deviations of the promoter and enhancer activity boost indices, respectively.

Activity balance index calculation:

$$\mathrm{Bal}=Z_{\mathrm{Promoter}}-Z_{\mathrm{Enhancer}}$$

Where Bal is the activity balance index.

Functional classification:

Promoter-dominant: R > 1

Enhancer-dominant: R < −1

Balanced: −1 ≤ R ≤ 1

### GC content calculation

GC content calculation:

$$\mathrm{GC}\;\mathrm{content}\;(\%)=\left(\frac{\;{\mathrm{Count}\;}^{’}G^{’}\;+\;{\mathrm{Count}\;}^{’}C^{’}}{\;\mathrm{Total}\;\mathrm{bases}\;}\right)\times 100$$

Where:

Count 'G'and'C' is the total number of guanine and cytosine bases in the sequence and Total bases is the total length of the sequence (including all bases A, T, G, and C).

### Change boost index calculation

Δboost index:

$$\Delta BI=BI_{Mut}-BI_{WT}$$

Where:

${BI}_{\mathrm{WT}}$ and ${BI}_{\mathrm{Mut}}$ are the wild-type and mutant boost indices, respectively.

### Activity ratio calculation

Activity ratio calculation:

$$R=BI_{\mathrm{Promoter}\;}-BI_{\mathrm{Enhancer}\;}$$

Where R is the activity ratio and ${BI}_{\mathrm{Promoter}}$ and ${BI}_{\mathrm{Enhancer}}$ are the promoter and enhancer boost indices, respectively.

### PRO-cap/seq data analysis

PRO-cap signal was defined as the total number of UMI-deduplicated read counts within the boundaries of tested elements. PRO-seq signal was defined as either 1, the total number of UMI-deduplicated read counts 100 (**Supplementary Fig. 1a**), 250 (**Supplementary Fig. 1b**), or 500 (**Supplementary Fig. 1c**) bp downstream (for forward elements) and upstream (for reverse elements) from the boundaries of tested elements, 2, the mean number of UMI-deduplicated read counts 100 (**Supplementary Fig. 1d**), 250 (**Supplementary Fig. 1e**), or 500 (**Supplementary Fig. 1f**) bp downstream and upstream from the boundaries of tested elements, 3, the total number of UMI-deduplicated read counts within the boundaries of tested elements including a 100 (**Supplementary Fig. 1g**), 250 (**Supplementary Fig. 1h**), or 500 (**Supplementary Fig. 1i**) bp extension downstream (for forward elements) and upstream (for reverse elements) from the boundaries of tested elements, or 4, the mean number of UMI-deduplicated read counts within the boundaries of tested elements including a 100 (**Supplementary Fig. 1j**), 250 (**Supplementary Fig. 1k**), or 500 (**Supplementary Fig. 1l**) bp extension downstream and upstream from the boundaries of tested elements.

### SuRE, lentiMPRA, ATAC-STARR-seq, and WHG-STARR-seq data analysis

To benchmark QUASARR-seq against orthogonal assays, we compared elements with at least a 10% reciprocal overlap also tested in SuRE, and at least a 50% reciprocal overlap also tested in lentiMPRA, ATAC-STARR-seq, and WHG-STARR-seq. Active enhancer calls for lentiMPRA, ATAC-STARR-seq, and WHG-STARR-seq were obtained using the same uniform active element call pipeline as described.

## Extended Data

## Data Availability

QUASARR-seq data will be made available in the ENCODE portal (www.encodeproject.org). PRO-cap (accession no. ENCSR220XSM) data were retrieved from the ENCODE portal. PRO-seq data will be made available in the ENCODE portal. Processed SuRE data were obtained from van Arensbergen et al.^28^ lentiMPRA (accession no. ENCSR382BVV), ATAC-STARR-seq (accession no. ENCSR312UQM), and WHG-STARR-seq (accession no. ENCSR661FOW) data were retrieved from the ENCODE portal. All accession codes not provided will be made available prior to publication.
